# Surgical treatment for pelvic lipomatosis using a bladder-sparing technique: A STROBE-compliant study

**DOI:** 10.1097/MD.0000000000016198

**Published:** 2019-06-28

**Authors:** Liyuan Ge, Xiaojun Tian, Guojiang Zhao, Jing Ma, Yimeng Song, Feilong Yang, Shudong Zhang, Lulin Ma

**Affiliations:** a Department of Urology; b Department of Ultrasonography, Peking University Third Hospital, Beijing, PR China.

**Keywords:** bladder-sparing surgery, pelvic fat mass extirpation, pelvic lipomatosis, ureteral reimplantation

## Abstract

The aim of this study was to report the experience and long-term efficacy of a novel surgical treatment for pelvic lipomatosis (PL) using a combination of pelvic fat mass extirpation and ureteral reimplantation.

Data of 8 patients with PL who underwent pelvic fat mass extirpation and ureteral reimplantation at our hospital from September 2010 to March 2018 were retrospectively reviewed. Demographics, serum creatinine level, radiographic changes, perioperative complications, and patient-reported outcomes were evaluated.

Surgeries were performed successfully without severe perioperative complications in all 8 patients. Median operating time was 150 minutes with a median estimated blood loss of 75 mL. Patients were discharged after a median of 8.5 postoperative days. Imaging studies at the first follow-up revealed varying extents of alleviation of hydronephrosis and 3 patients’ urinary symptoms were gradually relieved after surgery. During a median follow-up of 48.5 months (range, 10–100 months), all patients exhibited excellent surgical outcomes without evidence of disease progression, except 1 patient who underwent radical cystectomy with Bricker ileal conduit surgery due to hydronephrosis recurrence in the 49th postoperative month.

Based on these cases, pelvic fat mass extirpation and ureteral reimplantation is a safe and effective surgical treatment for PL.

## 1 Introduction

Pelvic lipomatosis (PL) is a rare disease characterized by excessive growth of normal adipose tissue around the rectum and pelvis.^[1,2]^ Its early symptoms are usually atypical and some patients are not diagnosed until severe upper tract obstruction or even kidney failure has occurred.^[3,4]^ Because of the ambiguous etiology, treatment options for PL are limited. Conservative treatment, such as dietary management, antibiotic agents, steroids, and radiation therapy, has demonstrated limited efficacy. Carpenter suggested that patients with PL could be separated into 2 clinical subgroups according to age. For older patients, the disease tends to be more indolent and may not progress for 10 years or longer. Thus, the main treatment goal for these patients should be to relieve lower urinary tract symptoms, which would involve relatively conservative surgical modalities, such as ureteral stent insertion and transurethral resection of the prostate. For younger patients, the disease, which is benign but aggressive, tends to result in progressive ureteral obstruction and require urinary diversion surgery, usually performed in the form of ilea conduit or ureterocutaneostomy.^[5]^ However, younger patients may not be willing to accept such treatment as either procedure may greatly affect their quality of life. With the development of surgical instruments and technique, some urologists have attempted to treat younger patients with bladder-sparing surgery and achieved impressive short-term outcome.^[6,7]^ However, there are no large-scaled and long-term data available for this technique. Thus, in this study, we evaluated the safety and long-term efficacy of pelvic fat extirpation and ureteral reimplantation, which could prove to be a novel treatment strategy for PL.

## 2 Methods

### 2.1 Study population

Following approval by the Medical Ethics Committee for Human Experiments (M2018183), we retrospectively searched the database for patients with PL who were treated at Peking University Third Hospital from September 2010 to March 2018. Written informed consent was obtained from all participants. Patients who underwent pelvic fat extirpation and ureteral reimplantation surgery were identified. Patients who received other therapies were excluded. Diagnosis of PL was confirmed by characteristic imaging manifestations and postoperative pathologic results. Clinical information extracted included age at surgery, sex, symptoms at diagnosis, examination results, body mass index (BMI), comorbidities, and pathologic results.

As some of the patients underwent postoperative serum creatinine examinations at other hospitals, to avoid bias caused by different reference ranges, we extracted the results of the final creatinine examination during hospitalization for analysis.

### 2.2 Surgical procedures

**Figure 1:**
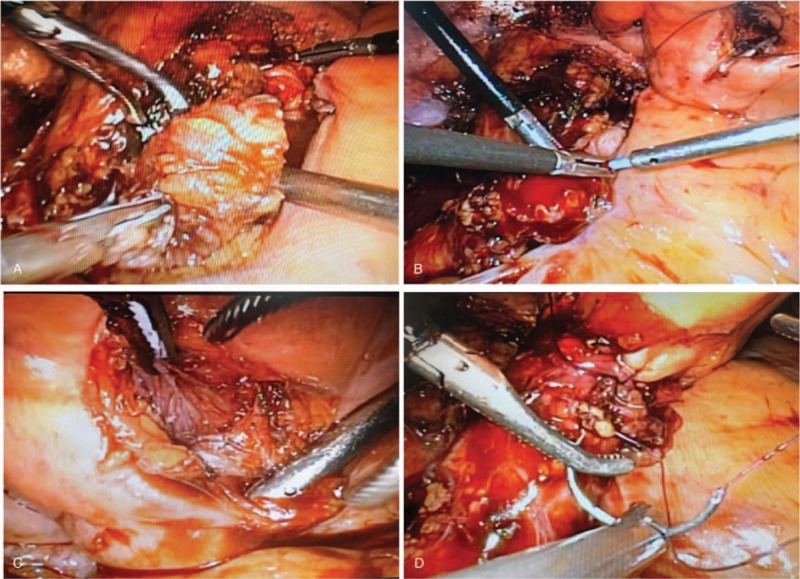
A, Separation of fat-coated bladder using ultrasonic knife, (B) separation of bladder muscularis and mucosa, (C) inserting double J stent into ureter, (D) reimplantation of ureter at the dome of bladder.

Surgical details are as follows. For laparoscopic surgery, after anesthesia, the patient was placed in the supine position. An incision was made at the midpoint between the xiphoid and umbilicus to insert a 12-mm trocar to establish CO_2_ pneumoperitoneum. Subsequently, bilateral (10 mm) ports were inserted under monitoring at the lateral border of the rectus abdominis at the umbilical level, whereas another bilateral (5 mm) ports were inserted 5 cm inside the upper iliac spine. To identify the right ureter, the right posterior peritoneum was opened and the fat was carefully dissected around the right external iliac vessels. Upon finding the right ureter, the middle and lower segments down to the bladder were carefully loosened. The left ureter was freed in the same way. Proliferated fat around the ureter and bladder was extirpated and sent for pathologic examination (Fig. 1A). Bilateral ureters were dissected at the bladder junction and then the bladder incisions were closed using 3–0 absorbable sutures. Two new incisions were made at the bladder dome to partially insert double-J stents into the bladder lumen (Fig. 1B and C). The ends of the dissected ureters were trimmed and then the other ends of the double-J stents were inserted into them. Then, bilateral ureters were reimplanted at the bladder dome (Fig. 1D). For open surgery, the only difference was that a midline incision was made to enter the abdominal cavity, whereas the other surgical methods were the same.

### 2.3 Postoperative treatment and follow-up

Patients underwent computed tomography (CT) to evaluate the surgical outcome 3 months after surgery, and outpatient surgery was performed to remove bilateral ureter stent tubes. Patients were asked to undergo urinary system ultrasound, magnetic resonance imaging, or CT to evaluate patency of the urinary system every 3 months within the first year and every 6 months thereafter. Serum creatinine level was measured to evaluate renal function during each follow-up. The Clavien-Dindo classification was used to evaluate the level of surgical complications.

### 2.4 Statistical analysis

Because of the relatively small sample size, continuous variables were presented as median and range.

## 3 Results

**Figure 2:**
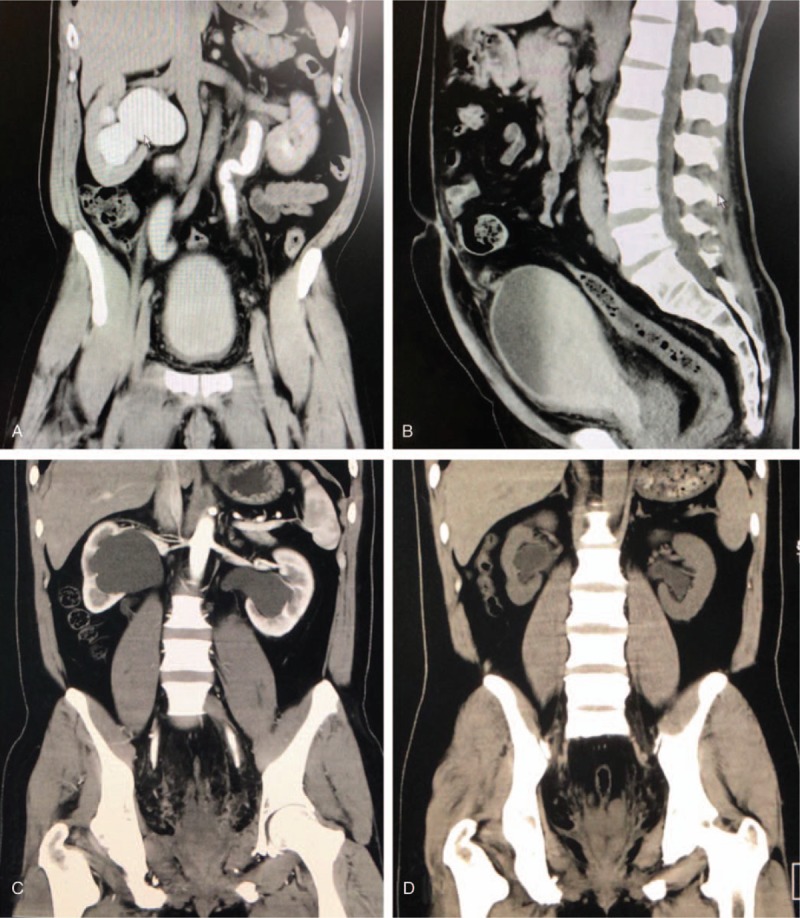
A, B, Pelvic computed tomography (CT) scans in coronal and sagittal planes showed that the bladder and sigmoid colon are compressed by an unencapsulated homogeneous soft tissue mass. C, D, Preoperative and postoperative CT scans of the same patient showed alleviation of bilateral hydronephrosis.

All 8 patients were men and of Han nationality. The median age was 47 years (range, 32–59 years) and the median BMI was 26.0 kg/m^2^ (range, 20.1–32.1 kg/m^2^). Three patients visited our hospital for lower urinary symptoms, including urinary frequency, dysuria, and urgency; the other 5 patients were found to have bilateral hydronephrosis incidentally during physical examination. Preoperative serum creatinine levels of 2 patients with hydronephrosis were slightly elevated (Table 2, case 3: 137 μM and case 4:132 μM; reference range: 53–130 μM), indicating potential impairment of renal function. All other patients’ preoperative serum creatinine levels were within normal range according to our hospital's reference range (53–130 μmoI/L). All patients underwent CT before surgery and the common radiologic characteristics were excessive pelvic fat deposition, alteration of bladder shape, and varying degrees of hydronephrosis (Fig. 2A and B). General characteristics are listed in Table 1.

**Table 1:**
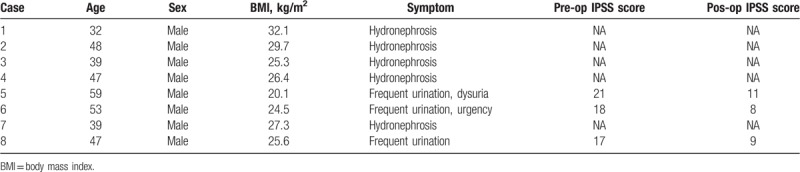
Patients characteristics.

Surgery was performed successfully in all 8 patients. Three patients underwent open surgery, 4 underwent laparoscopic surgery, and 1 (case 6) underwent conversion to open surgery intraoperatively due to severe pelvic fat adhesion. The median operating time was 150 minutes (range, 135–230 minutes) and the median estimated blood loss was 75 mL (range, 50–120 mL); no patient received blood transfusion. The drainage tube was indwelled for 4 to 15 days after surgery (median, 7 days). Early postoperative complications occurred in 3 cases, which included fever (case 6, Clavien-Dindo grade II, medical treatment) and lymphorrhagia (cases 5 and 6, Clavien-Dindo grade I, conservative treatment). All complications were handled properly and patients were discharged after a median of 8.5 postoperative days (range, 6–16 days).

Postoperative pathology showed fibrous connective adipose tissue. Postoperative serum creatinine levels of 2 patients with possible renal function impairment returned to normal range after surgery. Although some of the patients experienced slightly elevated serum creatinine levels postoperatively, the values were still within normal range; this was possibly related to surgical stress response. At the first postoperative follow-up, imaging studies revealed varying degrees of alleviation of hydronephrosis (Fig. 2C and D). Results by the phone call following-up, urinary frequency is the most improved symptom and postoperative International Prostate Symptom Score (IPSS) score also showed a decline in all the 3 patients (Table 1). Patient 5, who presented with preoperative dysuria and urinary frequency, reported a great relief of urinary frequency, whereas dysuria symptom only relieved partially and exists until the last follow-up. During a median follow-up of 48.5 months (range, 10–100 months), 1 patient (case 1) had hydronephrosis recurrence in the 49th postoperative month and underwent Bricker ileal conduit surgery. The other patients showed no evidence of recurrence. Perioperative parameters and outcomes are summarized in Table 2.

**Table 2:**
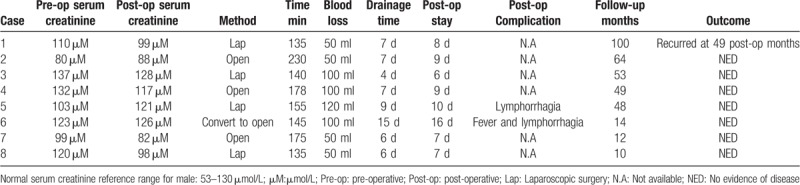
Perioperative and postoperative outcomes.

## 4 Discussion

PL is considered a benign progressive disease. Urinary diversion surgery is recommended for young patients, which may greatly affect quality of life.^[8]^ In the present study, we reported encouraging long-term outcomes of bladder-sparing surgery for treatment of PL. This procedure, however, remains challenging and larger, long-term studies should be conducted before applying this technique in more patients.

According to statistical data from the United States, between 1967 and 1975, the incidence of PL was approximately 0.6 to 1.7 per 100,000 hospital admissions.^[8]^ Clinical symptoms vary due to compression over different sites, such as the urinary tract, rectum, and even vascular system.^[9]^ As early atypical symptoms can resemble the features of benign prostatic hyperplasia, some elderly male patients may be misdiagnosed. Thus, the incidence is probably underestimated. Plain radiography, intravenous urography, CT, and magnetic resonance imaging have been reported to be of great diagnostic value.^[10]^ Our center also reported comparable diagnostic efficacy of a multimodal sonographic technique.^[11]^

Alteration of bladder shape is widely accepted as a valuable characteristic indicative of PL.^[12]^ Excessive pelvic fat accumulation in the perivesical and perirectal regions is, however, more common than alteration of bladder shape in the early stage of the disease. For patients in the late stage, alteration of bladder shape is often accompanied by hydronephrosis. Clinically, diagnosis of PL is made based on characteristic imaging manifestations after excluding other pelvic diseases, including teratoma, pelvic lipoma, retroperitoneal fibrosis, and pelvic liposarcoma.^[13]^ Although the radiographic appearances of teratoma and pelvic lipoma are similar to PL, a complete capsule with clear borders and without bladder shape alteration can be used for the differential diagnosis. Widespread retroperitoneal lesions without fat features on CT is the main difference between retroperitoneal fibrosis and PL. Liposarcoma can be differentiated based on systemic symptoms, such as rapid progression, early metastasis, wide range of lesions, and direct invasion of surrounding organs rather than compression.^[14]^

The etiology and natural course of PL remain unknown, but several studies have suggested that PL is possibly related to obesity.^[15,16]^ However, in our study, only 2 cases presented with definitive obesity (cases 1 and 2) and the BMI of our patients ranged from 20.1 to 32.1 kg/m^2^, which does not largely exceed the normal standard. Using a sequencing technique, PL adipose tissue has been found to demonstrate different epigenetic characteristics, indicating that methylation-regulating drugs may be the direction of future research.^[17]^ It has also been reported that approximately 70% of PL cases are associated with proliferative lesions of the bladder mucosa, such as cystic cysts and cystitis glandularis.^[18,19]^ Two patients (cases 5 and 6) with dysuria and urgency symptoms underwent cystoscopy biopsy before visiting our hospital; the pathologic results revealed cystitis glandularis. This finding highlights one of the concerns about this technique: Will patients convert to malignant disease when treated by organ-sparing surgery? Although the relationship between cystitis glandularis and bladder carcinoma remains unclear, some scholars believe cystitis glandularis is a precursor of bladder carcinoma.^[20]^ In our study, patients were closely followed and none converted to malignant disease at the last follow-up.

This disease typically occurs in patients between 20 and 60 years of age, with only 1 pediatric case being reported.^[21]^ Similarly, most of the patients in our cohort were young or middle aged. As various conservative modalities have been shown to be ineffective, surgical intervention may be the only method to abate the patient's symptoms or to retard disease progression.^[13]^ Surgical interventions include urinary diversion, transurethral resection of the prostate/bladder neck, and ureteral stent tube insertion. Ureteral stent tube insertion is the least invasive procedure but requires the stent tube to be changed regularly and may cause other complications, such as stones. For transurethral resection of the prostate/bladder neck, the procedure seems to have a good effect in only a certain portion of patients. Urinary diversion is the most effective treatment but also the most invasive.

Considering life expectancy and future quality of life, it is difficult for young patients to accept bladder resection for a benign disease. Recently, several successful attempts adopting a bladder-sparing technique have been reported. Halachmi et al^[6]^ reported the first case treated by pelvic fat excision and ureteral reimplantation using an ultrasonic-assisted lipectomy device. In another study, Ali et al^[7]^ resected the pelvic fat to preserve the bladder in a laparoscopic way. By removing the excessive pelvic fat, the bladder pressure can be reduced and the bladder capacity restored, which may alleviate the patient's symptoms during the storage period. On the contrary, the bladder neck stretching degree can also be reduced, which may alleviate the patient's symptoms during urination. However, neither of these studies reported the long-term efficacy.

Each surgical method has its own advantages and disadvantages and so far, there is no consensus on the optimal treatment. It has recently been reported that urodynamic study parameters can be useful for outcome stratification. Patients with PL presenting latter-half-section obstruction or bladder outlet obstruction features on urodynamic study suggests that the disease is more serious and have a higher risk of progression.^[22]^ In the present study, most of the patients had normal renal function (only 2 patients with hydronephrosis had slightly elevated serum creatinine levels before surgery), indicating that they were still at a relatively early stage. As PL progresses slowly and the patients were relatively young, it seemed worthwhile to attempt bladder-preserving surgery. After discussing all of the possible treatment options, the 8 patients in this cohort preferred bladder-sparing surgery with the hope of preserving quality of life. This highlights another concern about this technique: As the abnormal fat tissue continues to overgrow, how long will the surgical effect be sustained? In our cohort, during a median follow-up of 48.5 months, only 1 patient showed recurrence in the 49th postoperative month. In addition, we obliquely trimmed the ureters to increase the caliber of the outflow tract and reimplanted the ureters at the bladder dome, which can effectively decrease the possibility of being recompressed and prolong the time to relapse.

The last concern of this surgery is perioperative complications, such as ureteral damage and hemorrhage. The technical difficulty may be increased by fat adherence, lack of surgical cleavage planes, and hypervascularity of the process. Currently, with the emergence of laparoscopic devices, which may even be applied in open surgery, pelvic surgeries have become much easier than they were in the 1990s. In our study, all surgeries were performed by surgeons who had completed >200 laparoscopic pelvic surgeries. All of the surgeries were completed successfully and no patient experienced severe postoperative complications. Following our experience with this technique, we provide 4 important points of advice: When it comes to removing the fat tissue, you cannot be too careful, as proliferated pelvic fat is tough and rich in blood supply. Sometimes converting to open surgery is a wise choice when the anatomical exposure is incomplete or the anatomical landmarks are difficult to identify; It is difficult to separate the ureters from the adhesive fat tissue. Do not excessively strip the lower part of the ureters, so as not to affect the blood supply, which is important to prevent ureter damage; As pelvic fat is likely to regenerate, trimming the ureters obliquely and implanting them at the bladder dome may reduce the risk of recompression of the ureterovesical junction; and Lymphorrhagia may be one of the most common complications of this technique, as lymph circulation is devastated during fat excision. Using an ultrasonic scalpel to effectively seal the microvasculars can prevent lymphorrhagia, while leaving the drainage tube in place is beneficial for postoperative observation and possible conservative treatment.

There have been < 150 cases of PL reported worldwide. To date, this is one of the largest single-center clinical reports of PL treated by bladder-sparing surgery. There are some limitations, however. First, the sample size was small due to the low incidence of PL. Second, the study had a retrospective design and as such, the results should be confirmed in a larger prospective setting. Third, the follow-up time was relatively short and thus, a longer-term observation should be conducted.

## 5 Conclusions

To summarize, although the combination of pelvic fat mass extirpation and ureteral reimplantation achieved satisfactory outcomes, it should only be recommended for selected patients and performed by experienced surgeons due to its technical complexity. The experience with this technique remains limited and large-scale trials are required.

## Acknowledgment

The authors thank Yi Huang, Xiaofei Hou, Guoliang Wang (Department of Urology, Peking University Third Hospital) for providing patients’ information. The authors thank Kaiwen Ni (Department of Epidemiology and Hygienic Statistics) for helping us with statistical analysis. The authors also thank Min Lu (Department of Pathology, Peking University Third Hospital) for providing pathology results.

## Author contributions

**Conceptualization:** Xiaojun Tian, Lulin Ma.

**Data curation:** Feilong Yang.

**Formal analysis:** Guojiang Zhao.

**Funding acquisition:** Yimeng Song, Shudong Zhang.

**Resources:** Jing Ma.

**Writing – original draft:** Liyuan Ge, Xiaojun Tian.

**Writing – review and editing:** Shudong Zhang.

Lulin Ma orcid: 0000-0002-9283-5931.

## References

[1] Engels EP Sigmoid colon and urinary bladder in high fixation: roentgen changes simulating pelvic tumor. Radiology 1959;72:419–22. 13634406 10.1148/72.3.419

[2] Fogg LB Smyth JW Pelvic lipomatosis: a condition simulating pelvic neoplasm. Radiology 1968;90:558–64. 5642294 10.1148/90.3.558

[3] Miglani U Sinha T Gupta SK Rare etiology of obstructive uropathy: pelvic lipomatosis. Urol Int 2010;84:239–41. 20215833 10.1159/000277606

[4] Baas W O’Connor B El-Zawahry A Bilateral hydronephrosis and acute kidney injury secondary to pelvis lipomatosis. Can J Urol 2018;25:9217–9. 29524979

[5] Carpenter AA Pelvic lipoivlactosis: successful surgical treatment. J Urol 1973;110:397–9. 4742176 10.1016/s0022-5347(17)60231-3

[6] Halachmi S Moskovitz B Calderon N The use of an ultrasonic assisted lipectomy device for the treatment of obstructive pelvic lipomatosis. Urology 1996;48:128–30. 8693634 10.1016/s0090-4295(96)00100-8

[7] Ali A Swain S Manoharan M Pelvic lipomatosis: bladder sparing extirpation of pelvic mass to relieve bladder storage dysfunction symptoms and pelvic pain. Cent European J Urol 2014;67:287–8. 10.5173/ceju.2014.03.art15 PMC4165674 25247089

[8] Ali AS Hayes MC Birch B Health related quality of life (HRQoL) after cystectomy: comparison between orthotopic neobladder and ileal conduit diversion. Eur J Surg Oncol 2015;41:295–9. 24913090 10.1016/j.ejso.2014.05.006

[9] Locko RC Pelvic lipomatosis. JAMA 1980;244:1473–4. 7420639 10.1001/jama.244.13.1473

[10] Heyns CF Pelvic lipomatosis: a review of its diagnosis and management. J Urol 1991;146 (2 part 1):267–73. 1856914 10.1016/s0022-5347(17)37767-4

[11] Sun Y Wang J Chiang M Value of multimode sonography for assessment of pelvic lipomatosis compared with computed tomography. J Ultrasound Med 2016;35:1143–8. 27091913 10.7863/ultra.15.06053

[12] Zhang Y Wu S Xi Z Measuring diagnostic accuracy of imaging parameters in pelvic lipomatosis. Eur J Radiol 2012;81:3107–14. 22749803 10.1016/j.ejrad.2012.05.031

[13] Klein FA Smith MJ Kasenetz I Pelvic lipomatosis: 35-year experience. J Urol 1988;139:998–1001. 3361678 10.1016/s0022-5347(17)42744-3

[14] Andaç N Baltacioğlu F çimşit NÇ Fat necrosis mimicking liposarcoma in a patient with pelvic lipomatosis: CT findings. Clin Imaging 2003;27:109–11. 12639777 10.1016/s0899-7071(02)00519-3

[15] Sacks SA Drenick EJ Pelvic lipomatosis: effect of diet. Urology 1975;6:609. 1189150 10.1016/0090-4295(75)90512-9

[16] Craig WD Fanburg-Smith JC Henry LR Fat-containing lesions of the retroperitoneum: radiologic-pathologic correlation. Radiographics 2009;29:261–90. 19168848 10.1148/rg.291085203

[17] Xiong G He S Li X Whole genome epigenetic characteristics of pelvic lipomatosis: the new hope to prevent and cure this disease. Eur Urol Suppl 2018;17:e641.

[18] Heyns CF De Kock MLS Kirsten PH Pelvic lipomatosis associated with cystitis glandularis and adenocarcinoma of the bladder. J Urol 1991;145:364–6. 1988733 10.1016/s0022-5347(17)38342-8

[19] Tong RSK Larner T Finlay M Pelvic lipomatosis associated with proliferative cystitis occurring in two brothers. Urology 2002;59:602. 10.1016/s0090-4295(01)01609-0 11927334

[20] Smith AK Hansel DE Jones JS Role of cystitis cystica et glandularis and intestinal metaplasia in development of bladder carcinoma. Urology 2008;71:915–8. 18455631 10.1016/j.urology.2007.11.079

[21] Zaman W Singh V Kumar B Pelvic lipomatosis in a child. Urol Int 2002;69:238–40. 12372895 10.1159/000063935

[22] Chen Y Yang Y Yu W Urodynamic characteristics of pelvic lipomatosis with glandular cystitis patients correlate with morphologic alterations of the urinary system and disease severity. Neurourol Urodyn 2018;37:758–67. 28763116 10.1002/nau.23343

